# A dataset on patient-individual lymph node involvement in oropharyngeal squamous cell carcinoma

**DOI:** 10.1016/j.dib.2022.108345

**Published:** 2022-06-01

**Authors:** Roman Ludwig, Jean-Marc Hoffmann, Bertrand Pouymayou, Grégoire Morand, Martina Broglie Däppen, Matthias Guckenberger, Vincent Grégoire, Panagiotis Balermpas, Jan Unkelbach

**Affiliations:** a Departement of Radiation Oncology, University Hospital Zurich, Rämistrasse 100, 8091, Zurich, CH; b Head and Neck Tumor Center, Comprehensive Cancer Center Zurich, Rämistrasse 100, 8091, Zurich, CH; c Radiation Oncology Departement, Centre Léon Bérard, 28 Rue Laennec, 69008, Lyon, FR

**Keywords:** Head & neck squamous cell carcinoma, Oropharynx, Patterns of progression, Lymphatic involvement, Interface

## Abstract

**Dataset:**

We provide a dataset on lymph node level (LNL) involvement in 287 patients with newly diagnosed oropharyngeal squamous cell carcinoma (OPSCC). For each patient, ipsilateral and contralateral LNL involvement for levels I to VII is reported together with clinicopathological factors including TNM-stage, primary tumor subsite, tumor lateralization, HPV status, sex, age, smoking status, and primary treatment. LNL involvement was assessed individually based on available diagnostic modalities (PET, MRI, CT, fine needle aspiration) by reviewing pathology and radiology reports together with the radiological images. The data is shared as a CSV-table with rows of patients and columns of patient/tumor-specific information and the involvement of individual LNL based on the respective diagnostic modalities.

**Reuse potential:**

Patterns of lymphatic progression have never been reported on a patient-individual basis in as much detail as provided in this dataset. The data can be used to build quantitative models for lymphatic tumor progression to estimate the probability of occult metastases in LNLs. This may in turn allow for further personalization of the elective clinical target volume definition in radiotherapy and the extent of neck dissection for surgically treated patients. The data can be pooled with other data to build large multi-institutional datasets on lymphatic metastatic progression in the future.

**Co-submission:**

This paper supports the original scientific article by Roman Ludwig, Jean-Marc Hoffmann, Bertrand Pouymayou, Grégoire Morand, Martina Broglie Däppen, Matthias Guckenberger, Vincent Grégoire, Panagiotis Balermpas, Jan Unkelbach, “Detailed patient-individual reporting of lymph node involvement in oropharyngeal squamous cell carcinoma with an online interface”, Radiotherapy & Oncology [1]

## Specifications Table

**Table:** 

Subject	Oncology
Specific subject area	Quantification of lymphatic metastatic progression in oropharyngeal squamous cell carcinoma
Type of data	Table
How the data were acquired	We included patients with newly diagnosed HNSCC in the oropharynx treated at our institution between 2013 and 2019. The underlying data was acquired in routine clinical care and consists of electronic clinical reports, radiological images from computed tomography (CT), magnetic resonance imaging (MRI) and positron emission tomography (PET), as well as pathology reports from fine needle aspiration (FNA), biopsies and neck dissections.
Data format	Raw
Description of data collection	The acquired data was retrospectively analyzed by two radiation oncologists who extracted the relevant patient and primary tumor characteristics. Metastatic involvement of LNLs was assessed based on predefined criteria as described in “Experimental design, materials and methods” by reviewing pathology and radiology reports together with the radiological images.
Data source location	• Institution: University Hospital Zurich • City/Town/Region: Zurich • Country: Switzerland • Latitude and longitude: 47.37679270649676, 8.54915452425861
Data accessibility	The data can be found in the GitHub repository ``rmnldwg/lydata'' (Link: https://github.com/rmnldwg/lydata) inside the folder ``2021-usz-oropharynx''. A snapshot of the repository has been made persistent on Zenodo (Link: https://doi.org/10.5281/zenodo.6024778). The dataset can also be downloaded from our web-based GUI:https://2021-oropharynx.lyprox.org
Related research article	Roman Ludwig, Jean-Marc Hoffmann, Bertrand Pouymayou, Grégoire Morand, Martina Broglie Däppen, Matthias Guckenberger, Vincent Grégoire, Panagiotis Balermpas, Jan Unkelbach, “Detailed patient-individual reporting of lymph node involvement in oropharyngeal squamous cell carcinoma with an online interface”, Radiotherapy & Oncology, 169:p1–7, 2022 [1]

## Value of the Data

The general directions of lymph drainage in the neck are understood and the prevalence of involvement has been reported in the literature [2], [3], [4], [5]. However, further details of progression patterns in oropharyngeal HNSCC are insufficiently reported. For example, how the risk of metastases in one LNL depends on the involvement of other levels, T-category, HPV-status, and primary tumor lateralization is poorly quantified. As such, the value of the data reported is:

• Due to the detailed patient-individual reporting, the data can be used to generate hypotheses regarding the dependencies between the involvement of different LNLs and clinicopathological factors.
• The data may also be used to develop and train statistical models predicting the lymphatic progression of oropharyngeal head & neck cancer [6,7].
• This publication together with the online interface to view the data may motivate other researchers in the field to share their data in similar detail. It may therefore provide the basis for collecting large multicenter datasets of lymphatic progression patterns.
• Eventually, better quantification of the risk of LNL involvement may allow for further personalizing CTV-N definition for radiotherapy based on an individual patient's state of disease progression at the time of diagnosis. This may lead to volume-deescalated treatment strategies for selected patients, potentially reducing toxicity.

## 1 Data Description

### 1.1 Data

The data is provided as a CSV-table containing one row for each of the 287 patients. The table has a header with three levels that describe the columns. Below we explain each column in the form of a list with three levels.

1) patient: General information about the patient's condition can be found under this top-level header.
  a) #: The second level under patient has no meaning and exists solely as a filler.
    i) id: Enumeration of the patients
    ii) sex: Sex of the patient
    iii) age: Patient's age at diagnosis
    iv) diagnose_date: Date of diagnosis (format YYYY-mm-dd) defined as the date of first histological confirmation of HNSCC.
    v) alcohol_abuse: true for patients who stated that they consume alcohol regularly, false otherwise
    vi) nicotine_abuse: true for patients who have been regular smokers (>10 pack years)
    vii) hpv_status: true for patients with human papilloma virus associated tumors (as defined by p16 immunohistochemistry)
    viii) neck_dissection: Indicates whether the patient has received a neck dissection as part of the treatment.
    ix) tnm_edition: The edition of the TNM classification used to stage the patient [8]
    x) n_stage: N-category of the patient according to the TNM classification used, describing the degree of spread to regional lymph nodes
    xi) m_stage: Indicating the presence of distant metastases according to the TNM classification.
2) tumor: Information about tumors is stored under this top-level header
  a) <number>: The second level enumerates the synchronous tumors. In our database, no patient has had a second tumor, but this structure of the file allows us to include such patients in the future. The third-level headers are the same for each tumor.
    i) location: Anatomic location of the tumor
    ii) subsite: ICD-O-3 code corresponding to the tumor location according to the world health organization [9, 10]
    iii) side: Lateralization of the tumor. Can be “left” or “right” for tumors that have their center of mass clearly on the respective side of the mid-sagittal line and “central” for patients with a tumor on the mid-sagittal line.
    iv) extension: True if part of the tumor extends over the mid-sagittal line
    v) volume: Volume of the tumor in cm^3^ (not recorded in our dataset)
    vi) stage_prefix: Prefix modifier of the T-category, which can be “c” for clinical staging and or “p” for pathological staging.
    vii) t_stage: T-category of the tumor, according to TNM staging
3) <diagnostic modality>: Each recorded diagnostic modality is indicated by its own top-level header. In this file FNA, CT, MRI, PET, path (pathology) and pCT (radiotherapy planning CT) are provided
  a) info:
    i) date: Day on which a diagnose with the respective modality was performed
  b) ipsi: All findings of involved lymph nodes on the ipsilateral side of the patient's neck
    i) <LNL>: One column is provided for each recorded lymph node level. For each level true indicates at least one lymph node was considered malignant in the respective LNL, false means no malignant lymph node has been found, and an empty field indicates that no diagnosis is available for this LNL and the respective diagnostic modality. <LNL> can be: I, Ia, Ib, II, IIa, IIb, III, IV, V, VI, VII, VIII, IX, X.
  c) contra: Same as b) but for the contralateral side of the patient's neck
    i) <LNL>: same as under 3)b)i)

Dates of diagnosis and diagnostic procedures were anonymized by shifting all dates for a patient by a random time offset, preserving the relative time difference between diagnosis and diagnostic procedures.

### 1.2 Online interface for data viewing

We provide a user-friendly and intuitive graphical user interface to view the dataset, which is available at https://2021-oropharynx.lyprox.org/. The GUI has two main functionalities: the *patient list* and the *dashboard*. The patient list allows for viewing the characteristics of a patient, corresponding to one row of the CSV file, in a visually appealing and intuitive way. The dashboard allows for filtering of the dataset. For example, the user may select all patients with primary tumors extending over the mid-sagittal plane with involvement of ipsilateral level III. The dashboard will then display the number or percentage of patients with metastases in each of the other LNLs.

### 1.3 Patient cohort characteristics

**Fig. 1:**
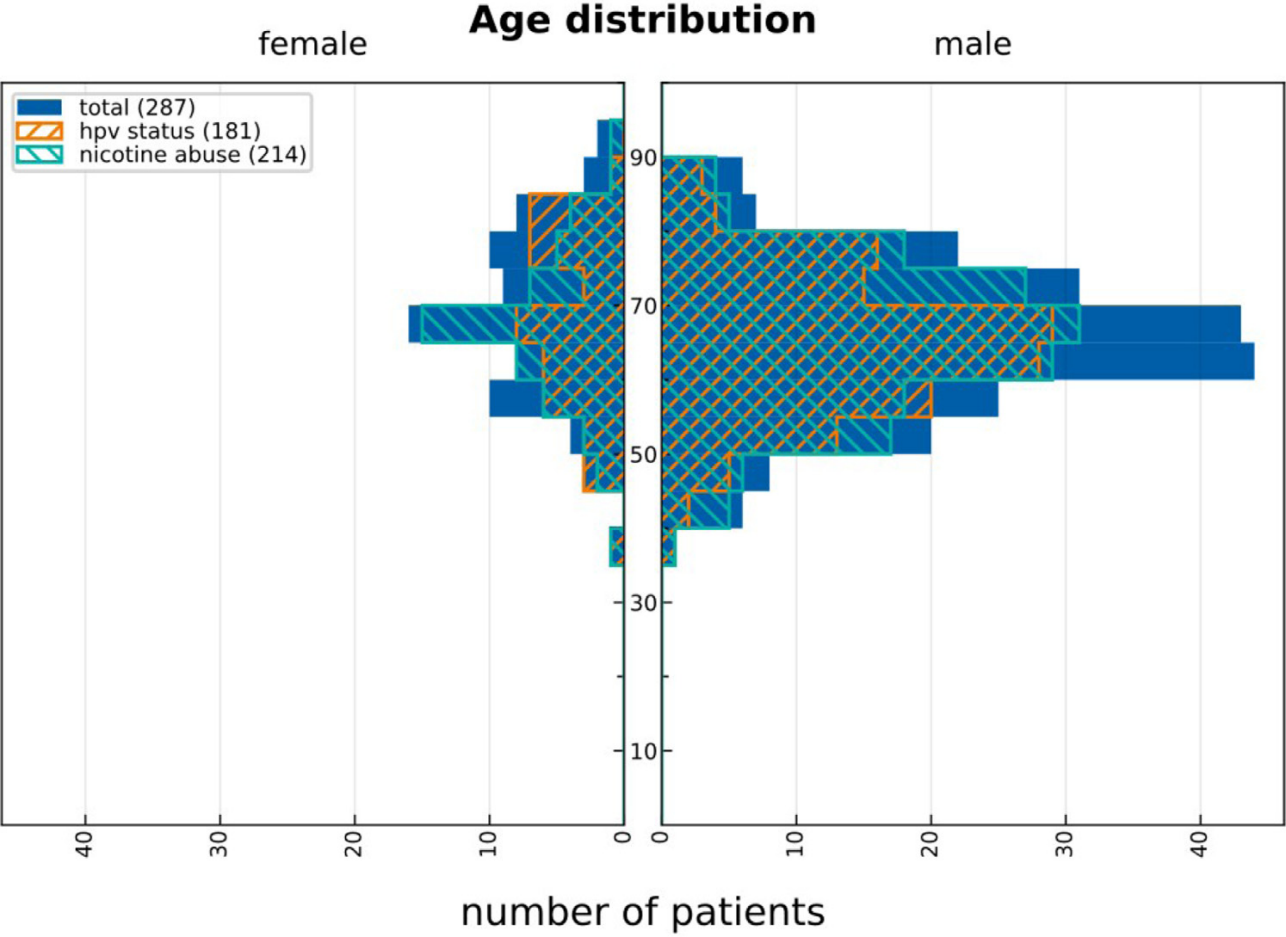
Age distribution of all patients (solid blue), all patients that are HPV positive (hatched orange) and all former/current smokers (hatched green).

Fig. 1 displays demographics of the cohort. 74.2% of the patients are male, 25.8% female. Median age is 65 years for male patients and 67 years for female patients. 74.6% of all patients reported to be former or current smokers. Among the 73 non-smokers, 67 had positive, 4 negative, and 2 unknown HPV status. Fig. 1 provides no indication that the age distribution of HPV positive patients is substantially different compared to the general patient population.

**Fig. 2:**
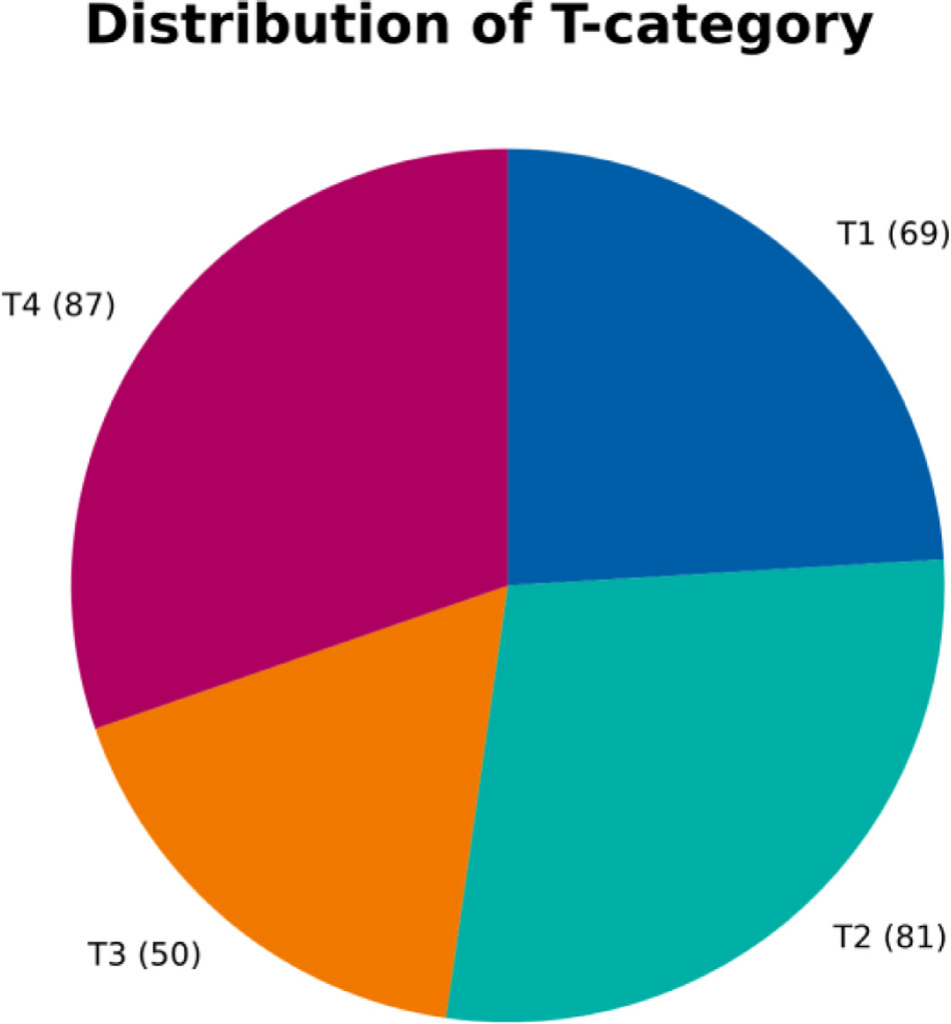
Pie chart displaying the relative portions of patients that presented with the respective T-categories.

Fig. 2 shows the distribution over the T-category of the primary tumor. The dataset contains patients treated with surgery only, postoperative radiotherapy, and definitive radiotherapy. Therefore, the dataset is likely representative of the general HNSCC patient population. Compared to other publications reporting lymph node involvement in surgically treated patients [3,4,11], our dataset contains a larger percentage of late T-category patients, who are typically treated with definitive chemoradiotherapy.

**Fig. 3:**
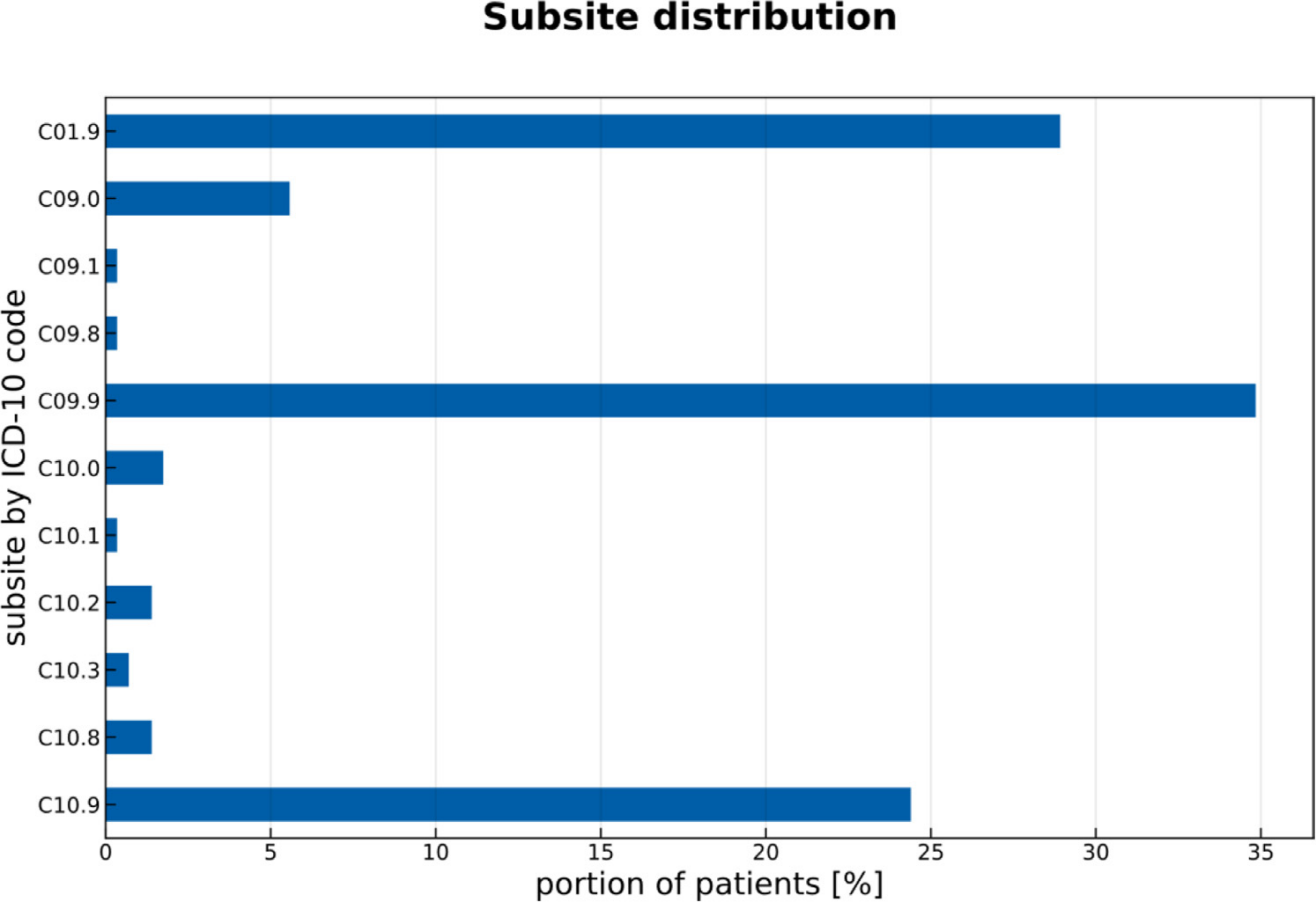
Distribution of primary tumor location categorized by ICD-O-3 code.

**Fig. 4:**
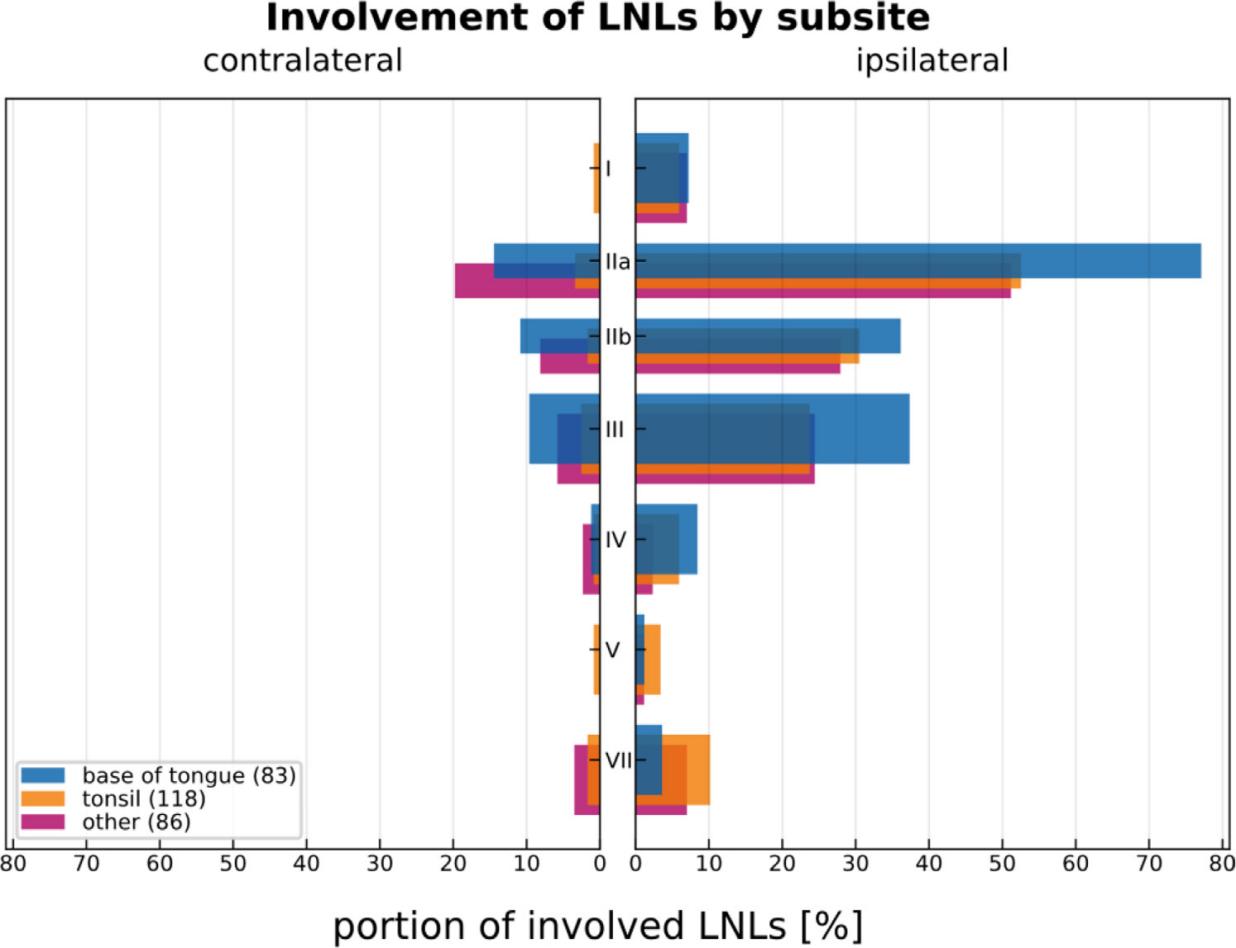
Distribution of involvement of individual lymph node levels, separated by primary tumor location (base of tongue (C01.9), tonsil (C09.0, C09.1, C09.8, C09.9) and other oropharyngeal regions).

Fig. 3 shows the distribution over the different primary tumor subsites identified by their respective ICD-O-3 code. Fig. 4 displays the prevalence of lymph node level involvement for primary tumors in different anatomical subsites. To generate Fig. 4, a lymph node level was considered involved if it was considered positive in one of the available diagnostic modalities. A detailed analysis of lymph node involvement is provided in the related research article [1].

## 2 Experimental Design, Materials and Methods

We reviewed patients treated for newly diagnosed oropharyngeal squamous cell carcinoma treated at the University Hospital Zurich (USZ) between 2013 and 2019. The resulting dataset of 287 patients contains patients treated with surgery alone, postoperative radiotherapy, or definitive radiotherapy. Main exclusion criterion was prior radiotherapy or surgery to the neck. Data collection was performed by 2 experienced radiation oncologists by reviewing radiology and pathology reports together with the diagnostic images.

### 2.1 Criteria for lymph node involvement

An LNL was considered involved if the main mass of at least one malignant lymph node was located within the level. Criteria for considering a lymph node as malignant followed the description in Biau et al [12] and were as follows:

• CT and MRI: Lymph nodes larger than 1 cm in the smallest transverse diameter were considered positive. In addition, all lymph nodes showing central necrosis and/or loss of fatty hilum were labeled positive.
• PET-CT/MR: Nodes with an SUV of 2.5 or more and fulfilling the above criteria regarding the underlying registered CT or MRI were considered positive. Additionally, nodes with an SUV of 2.5 or more not fulfilling the CT/MRI criteria above were also considered positive if not proven negative on FNA-derived cytology. Nodes without considerable FDG-uptake (SUV<2.5) were always considered negative.
• FNA: FNA was performed based on institutional practice. 235 patients had at least one node punctured. LNLs with no FNA performed were labeled 'unknown', indicated as blanks in the CSV, LNLs with positive findings were labeled positive. If the FNA findings were negative, the LNL was labeled healthy, even though only selected nodes in the level were punctured. Thus, in the interpretation of FNA involvement it must be taken into account that a negative result does not exclude the possibility of occult metastases in the LNL.
• radiotherapy planning CT: An LNL was considered positive if it contained the main mass of a lymph node contoured as nodal gross tumor volume (GTV-N), and thus may be considered a consensus decision originating from the available diagnostic modalities. For patients receiving radiotherapy following neck dissection, the planning CT is labeled negative for the resected levels.

### 2.2 Pathology after neck dissection

In case patients received a neck dissection, we recorded whether the histology showed signs of positive extra-nodal extension and how many malignant lymph nodes were resected. However, neck dissection was performed en bloc and not separated by level so that detailed information on the location of pathological lymph nodes was not available. Therefore, pathology findings are not contained in the results reported in this paper.

### 2.3 Primary tumor characteristics

Tumors extending over the midline were assigned to left/right according to the main primary tumor mass. When this was not possible, tumors were defined as 'central', in which case the side with more lymph node involvement was defined as ipsilateral.

Specific subsites (ICD-O-3 codes) of oropharyngeal cancer included the base of tongue, the tonsils as well as the oropharyngeal side of the vallecula and the posterior or lateral wall of the oropharynx. All tumors were assigned to one ICD-O-3 code. For extended primary tumors, this was done based on the location of the main mass. For visualization in the GUI, subsites were grouped into three categories: 1) Base of tongue (C01.9), 2) Tonsil (C09.0, C09.1, C09.8, C09.9), and 3) Other (C10.0, C10.1, C10.2, C10.3, C10.4, C10.8, C10.9). However, the detailed subsite information is contained in the CSV data base file and accessible via the patient list in the GUI.

## Ethics Statements

The research has been carried out in accordance with The Code of Ethics of the World Medical Association (Declaration of Helsinki). The retrospective data collection was approved by the cantonal ethics committee of Zurich (BASEC-No.: 2019–00684).

## CRediT Author Statement

**Roman Ludwig:** Software, Data Curation, Visualization, Writing – review & editing; **Jean-Marc Hoffmann:** Investigation, Data curation, Writing – review & editing; **Bertrand Pouymayou:** Software, Data Curation; **Martina Broglie Däppen:** Resources; **Grégoire Morand:** Resources; **Matthias Guckenberger:** Writing – review & editing; **Vincent Grégoire:** Writing – review & editing; **Panagiotis Balermpas:** Investigation, Methodology, Conceptualization, Writing – review & editing; **Jan Unkelbach:** Conceptualization, Methodology, Writing – original draft, Supervision, Project administration

## Declaration of Competing Interest

The authors declare that they have no known competing financial interests or personal relationships that could have appeared to influence the work reported in this paper.
